# A Fractional Creep Constitutive Model Considering the Viscoelastic–Viscoplastic Coexistence Mechanism

**DOI:** 10.3390/ma16186131

**Published:** 2023-09-08

**Authors:** Jia Zhao, Weigang Zhao, Kaize Xie, Yong Yang

**Affiliations:** 1 School of Civil Engineering, Shijiazhuang Tiedao University, Shijiazhuang 050043, China; 220190114@student.stdu.edu.cn; 2 School of Safety Engineering and Emergency Management, Shijiazhuang Tiedao University, Shijiazhuang 050043, China; zhaoweig2002@163.com (W.Z.); yangy@stdu.edu.cn (Y.Y.)

**Keywords:** constitutive models, Viscoelastic–Viscoplastic coexistence mechanism, fractional calculus, asphalt mixtures, creep test

## Abstract

In order to improve the accuracy and universality of the nonlinear viscoelastic-plastic mechanical behavior characterization method of asphalt mixture, a new criterion for the division of the creep process of materials was established based on the strain yield characteristics, and the coexistence mechanism of Viscoelastic–Viscoplastic strain was proposed in the subsequent yield phase; then, a viscoelastic element was constructed in the form of a parallel connection of two fractional viscoelastic elements based on fractional calculus theory, and its mathematical equations were derived; with novel viscoelastic elements, a constitutive model characterizing the whole creep process of asphalt mixtures was developed and its analytical expression was derived. The laboratory short-term creep test of Cement and Asphalt Mortar (CA mortar) and the simulation test data of asphalt mixtures from the references were used to verify the constitutive model. The results show that the creep constitutive model of asphalt mixture established in this paper has excellent fitting accuracy for different phases of the creep process of asphalt mixture under different stress levels, where the minimum fitting correlation values R^2^ for CA mortar, asphalt mixture (applied to pavement engineering), and asphalt sand are 0.9976, 0.981, and 0.979, respectively. Therefore, this model can be used to provide a theoretical reference for the study of the characterization of the mechanical behavior of asphalt materials.

## 1. Introduction

Due to the presence of asphalt matrix components in asphalt mixtures, which can provide excellent bonding and vibration reduction performance for engineering structures, they are widely used in engineering fields such as road pavement engineering and high-speed railway ballastless track engineering. However, the asphalt mixture exhibits complex time-varying mechanical behavior characteristics under long-term load, which poses a great challenge for structural design [1,2,3]. The establishment of a reasonable, accurate, and universal constitutive model of asphalt mixture has become a research hotspot in the field of engineering, which is of great significance for studying the evolution of the mechanical properties of materials and optimizing structural design methods.

As a typical viscoelastic-plastic material, the viscous mechanical behavior of asphalt mixture has a significant temporal correlation. For verifying and predicting the time-varying characteristics of the deformation of asphalt mixture under prolonged-term stress, the creep testing is an essential research measure [4,5,6,7]. According to the change characteristics of the whole creep process of asphalt mixture and the classification method of the creep process of viscoelastic materials in most studies, the creep process of asphalt mixture is divided into three phases: the decay creep phase, the stable creep phase, and the accelerated creep phase. For describing the strain changes of the creep process of the asphalt mixture, based on the total strain decomposition method, researchers usually use strain decomposition ideas to describe the different phases of the creep process of asphalt mixture using appropriate mechanical properties elements such as elastic, plastic, and viscoelastic or viscoplastic elements. With different combinations, constitutive models suitable for describing the stress–strain curve properties of the studied materials at different creep phases can be constructed. For instance, Zhang [8] developed a modified Burgers model to describe the nonlinear viscoelastic properties of rubber asphalt materials by concatenating one nonlinear modified viscous element, one elastic element, and one Kelvin model; Xiao [9] concatenated a nonlinear rheological element in series to the classical Burgers model to form a new constitutive model to describe the creep behavior of asphalt mixtures with large porosity; Yin [10] used the modified Burgers model, which combines the classical Burgers model with one Maxwell model and one fractional Kevin model in series, to analyze the dynamic creep performance of asphalt mixtures of the pavement. Xu [11] realized the study of transplanting the series Viscoelastic–Viscoplastic constitutive model to finite element calculation, and analyzed the mechanical behavior simulation and calculation of the asphalt pavement structure. Zhang [12] developed a creep model of asphalt mixture by using a three-element linear viscoelastic model in series with a viscoplastic element, and verified the model by repeated laboratory tests. During service, the creep behavior of the asphalt mixture is susceptible to load or environmental factors. Therefore, researchers have further carried out the creep model research of asphalt mixture under the influence of different conditions (temperature, stress level, water, etc.). An [13] carried out creep tests of asphalt mixtures under the influence of different temperatures and stress levels, and used a viscoplastic creep model in series with a nonlinear viscoplastic element and the Burgers model to achieve high-precision data fitting, and used this model to predict the creep behavior of asphalt mixtures under complex conditions. Zhou [14] used the Burgers model to characterize the viscoelastic behavior of asphalt mixture, studied the influence of two kinds of water effects, the freeze–thaw cycle and elevated-temperature water immersion, on the parameters of the creep model of asphalt mixture, and made a preliminary qualitative analysis of the law. Li [15,16] studied the influence of temperature and load stress on the creep deformation rate of asphalt mixture with different grades through static creep experiments, and used the Burgers model to characterize the viscoelastic energy of asphalt mixture, and analyzed the shift law of parameters in the model under different temperature and stress levels. The results show that the increase in temperature and stress level has a clear effect on the viscoelasticity of the asphalt mixture, with the temperature factor having more influence on the overall consistency of the model parameters. Zhao [17] analyzed the influence of viscoelastic short-term and long-term creep compliance of asphalt mixture under different confining pressure and temperature conditions. It can be seen that the creep test is an indispensable test method for constructing the constitutive model of asphalt mixture.

The construction methods for the creep constitutive model of asphalt mixture can be broadly divided into three categories. The first is the direct data fitting method, that is, according to the stress–strain data obtained from a static or quasi-static creep test of the asphalt mixture, after dimensionless processing, the geometric characteristics of the data curve are analyzed, and the appropriate mathematical function is selected for data fitting, and the creep constitutive model of the asphalt mixture is constructed. In this process, the quantitative relationship between the external factors and the coefficients of the chosen function can be analyzed and constitutive equations can be established to predict the mechanical behavior of asphalt mixtures under complex conditions [18,19,20]. However, while these methods can achieve higher fitting accuracy for test data, the constitutive equations established lack physical meaning and are difficult to apply to other types of asphalt mixtures. The second is to develop an appropriate constitutive model based on thermodynamics and the theory of internal variables, taking into account the dissipative mechanisms induced by irreversible displacements. This kind of method satisfies the basic laws of thermodynamics. By introducing a set of internal variables characterizing the internal structural changes of the material during viscoelastic deformation, the internal state of the material and the external response are linked through mathematical derivation and the irreversible energy dissipation process inside the material is realized. This approach has found numerous applications in the study of constitutive models of concrete materials that take into account performance degradation, and in the study of rheological constitutive models of rock materials. Currently, it is also being applied to the study of creep constitutive models for asphalt mixtures. This approach clearly expresses the shift of the internal state of matter away from logic and has a clear physical meaning. However, due to the unfixed potential function form of the thermodynamic state variables involved in the modeling process and the large number of internal variables, the mathematical derivation workload is heavy and most of the internal variables cannot be verified in the test, which makes it difficult to be widely promoted in practical applications [21,22,23,24]. The third (mechanical component combination) is widely used at present, which applies some mechanical components that can characterize the elasticity, plasticity, viscoelasticity, or viscoplasticity of the asphalt mixture, through a reasonable combination way, to establish the constitutive model that can adapt to the mechanical behavior of asphalt mixture at different phases of creep process. At first, most of these approaches were aimed at constructing constitutive models capable of characterizing the mechanical behavior of linear viscoelasticity [25,26]. As nonlinear problems became more and more prominent and intractable in the engineering field [27], some researchers began to study the nonlinear mechanical behavior of asphalt mixture under complex conditions through the measures of the nonlinear correction of the components in the viscoelastic constitutive model or the establishment of new elements adapted to nonlinear mechanical behavior combined with relevant nonlinear theories. In this regard, Zhang [28] introduced the strain equicalent principle of damage mechanics, establishing a type of nonlinear viscoelastic-plastic damage constitutive model. Zhang [29] considered the nonlinearity induced by the hardening and damage mechanisms of asphalt mixtures throughout the creep process and constructed a fractional creep damage model based on the fractional calculus theory to describe the complex viscoelastic mechanical behavior of asphalt mixtures in terms of fractional Abel elements. An [30] constructed a nonlinear viscoelastic-plastic creep model of the asphalt mixture with six mechanical parameters in series with the Burgers model and nonlinear viscoplastic elements, which can better describe the accelerated creep phase of asphalt mixture. Chen [31] used the Schapery model, the Findley model, and the multiple creep integral model to characterize the nonlinear viscoelastic properties of asphalt mixture. Liu [32] used the Weibull function to describe the distribution of internal defects in asphalt mixture, and introduced it into the nonlinear adhesive pot element of the modified Burgers model to establish the viscoelastic damage model of asphalt mixture. The results show that this model can fully reflect the creep characteristics of asphalt mixture in three phases. For another instance, the external factors affecting the material parameters related to asphalt mixture were introduced, and the nonlinear function (power function or exponential function) relationship between them and the material parameters of the constitutive model was constructed, so as to modify the material parameters in the established linear constitutive model, and realize the expression of the nonlinear viscoelastic mechanical behavior of the asphalt mixture constitutive model [33,34].

Recently, based on fractional order theory, a nonlinear viscoelastic constitutive model has been developed by scholars to characterize asphalt mixtures. Firstly, fractional mechanical components (such as the Abel adhesive pot) between linear elasticity and linear viscoelasticity has been developed by using fractional derivative theory, which realized the characterization of the nonlinear viscoelastic mechanical behavior of asphalt mixture. Then, according to the differences in mechanical behavior of materials with different types of components, and combining some linear mechanical elements, constitutive models suitable for the corresponding asphalt mixture were established [35,36,37]. However, most of the current models only achieve a characterization of the viscoelastic or viscoplastic mechanical behavior of a particular asphalt mixture, but do not uniformly characterize the mechanical and deformation behavior of a composite containing an asphalt matrix. On the one hand, this is related to the ability of constitutive models with different structural forms to characterize certain mechanical behaviors. On the other hand, this may also be related to the lack of uniform and accurate qualitative understanding of the deformation of the asphalt mixtures at different phases in the stress deformation process.

Motivated by the above discussion, in this paper, we establish a different criterion for dividing the creep process of asphalt mixtures according to the mechanical yield of the material, and propose a mechanism for the coexistence of viscoelastic and viscoplastic strains in the subsequent yield phase. In order to improve the generality of the viscoelastic characteristic, a special viscoelastic element consisting of two fractional viscoelastic elements in parallel has been proposed. Based on this, a constitutive model that characterizes the whole creep process of asphalt mixtures was developed. The correctness and applicability of the developed model is verified by short-term creep tests on CA mortar (one type of asphalt mixture) in the laboratory and test data on asphalt mixtures in the fitting reference example. The results can provide a new research perspective for analyzing the phase division of the creep development of asphalt mixture, which is different from traditional theories, and provide a new theoretical model reference for the subsequent study of the Viscoelastic–Viscoplastic mechanical behavior of asphalt mixture under the influence of damage and multiple factors.

## 2. Phases Division and Strain Property of Creep Process

**Figure 1:**
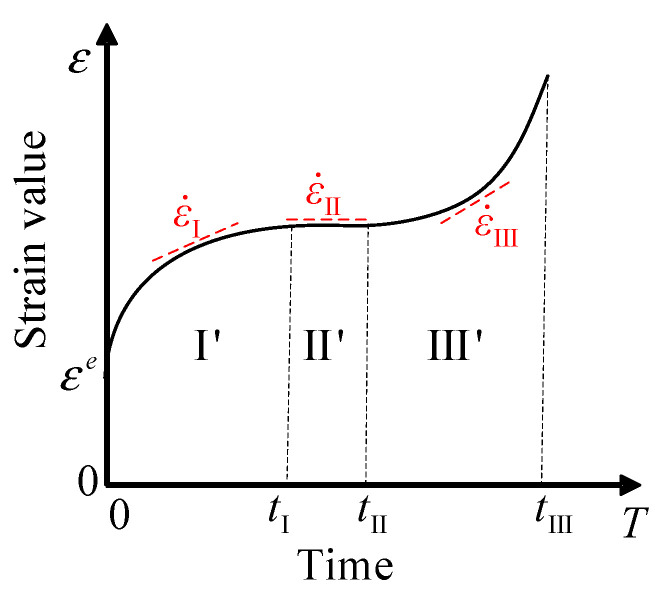
Classical three phases of creep.

**Figure 2:**
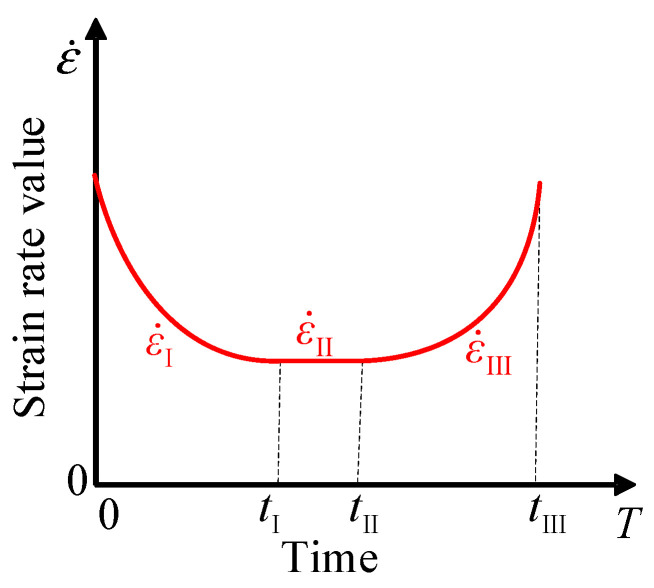
Variation of strain rate in three phases of creep.

The three phases of creep are mainly divided according to the different strain rate characteristics of the different phases, as shown in Figure 1 and Figure 2. In the decay creep phase, the strain rate gradually decreases from an initially high value. It enters the stable creep phase when the strain rate does not change. In the stable creep phase, the creep rate of the asphalt mixture remains essentially constant, and it enters the accelerated creep phase when the strain rate starts to increase. It is well known that the key to determining the dividing points of the different phases is the strain rate of the stable creep phase. When the strain rate and time curve are calculated with strain-time test data, the strain rate entering the stable creep phase is an approximation rather than a constant value. In this way, a judgment error will be introduced in determining the time points at which the different phases enter, resulting in an inaccurate constitutive model.

Moreover, it is known that, according to the creep test results of viscoelastic-plastic materials in literature, without considering the influence of temperature and loading rate, when the loading stress is large, the time of each creep phase, especially the time of the stable phase, will be sharply reduced, so the stable creep phase is not obvious, and even may not appear. However, most scholars conduct modeling studies by separately matching different creep phases with appropriate mechanical models. This well-established model does not effectively explain the disappearance of the stable creep phase.

Also, in some papers [24,30], the acceleration phase is described using a viscoplastic model. However, such a description directly restricts the strain properties of the accelerated creep phase to the viscoplastic strain, which lacks a criterion of discrimination.

**Figure 3:**
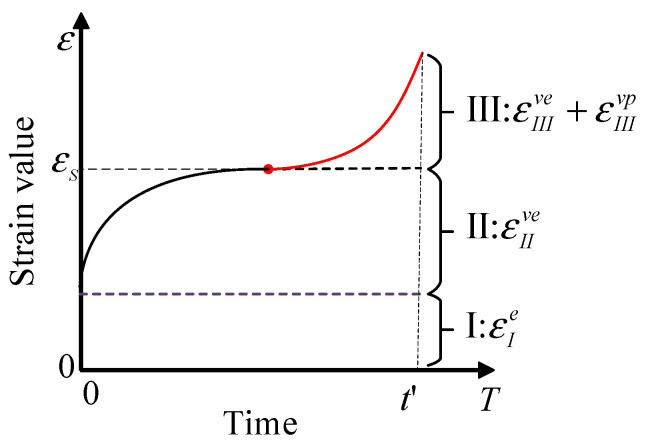
New three phases of creep and corresponding stain properties.

To this end, starting from the study of the strain properties in the whole creep process of viscoelastic-plastic materials in this paper, we divide the phases into the instantaneous elastic phase, the pre-strain yield phase, and the subsequent strain yield phase by using the strain yield values. The strain properties of the corresponding phases are elastic, viscoelastic, and viscoelastic-plastic strains, respectively. The judgment on the strain properties of the subsequent yield phase can be understood as follows: the stress–strain relation of the material during the loading process has a one-to-one correspondence, provided that the temperature and loading rate are constrained. However, the deformation of viscoelastic-plastic materials is time-dependent due to the presence of viscous effects. The mechanism of viscoelastic strain does not disappear immediately when the material undergoes yielding and plastic deformation. Instead, it persists under the influence of plastic deformation through a different mechanism than the viscoelastic deformation prior to yielding. This can be defined as the “inertia effect of viscoelastic deformation”. As a result, both viscoelastic and viscoplastic strains coexist during the subsequent yielding phase of viscoelastic-plastic materials. Therefore, the strain properties at different phases of the creep process will be shown in Figure 3.

## 3. A Fractional Viscoelastic Element

**Figure 4:**
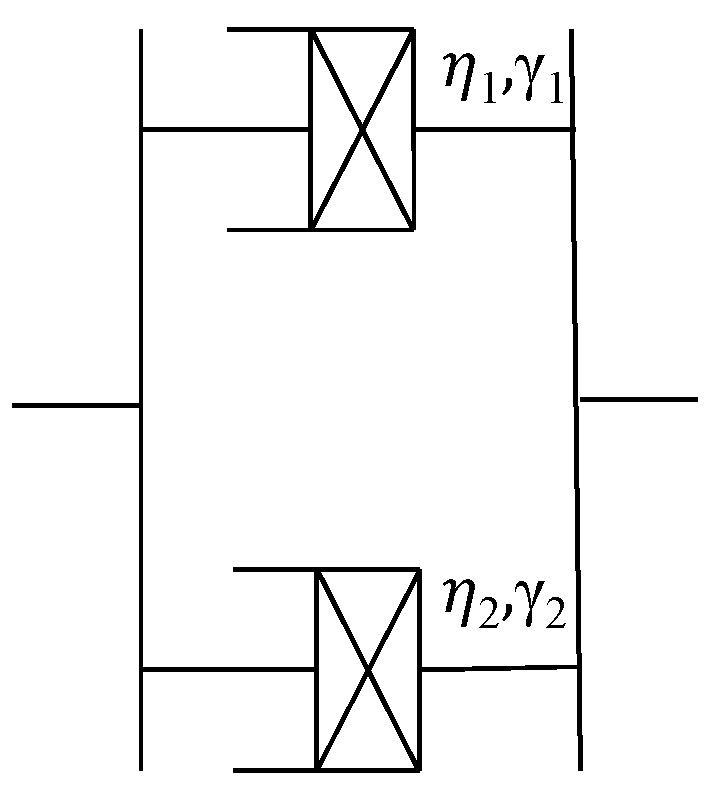
Fractional viscoelastic element.

Maxwell, Kelvin, and other linear models composed of Hooke elements and Newton elements in different combinations are constantly used to describe the viscoelastic mechanical behavior of materials. However, the viscoelastic deformation of asphalt mixtures at larger stresses exhibits pronounced nonlinear features. Therefore, based on the superiority of fractional calculus theory in describing nonlinear physical features, a different viscoelastic element with two fractional viscoelastic elements in parallel is proposed, as shown in Figure 4. *η_i_*, *γ_i_* represent the viscoelastic coefficient and derivative order of the viscoelastic element; the subscript *i* is the number of elements of the two fractional viscoelasticity.

According to the definition of the fractional derivative operator of the Riemann–Liouville type, the constitutive equation for the fractional viscoelastic element can be written as follows.

(1) $$\sigma =\eta \frac{d^{\gamma }\varepsilon }{dt^{\gamma }}$$

where *σ* and *ε* are stress and strain respectively; *t* represents time.

When *γ* = 0, Equation (1) can be simplified as *σ* = *ηε*, which shows the time-independent features of the Hooke element; when *γ* = 1, Equation (1) becomes $\sigma =\eta \dot{\varepsilon }$, showing that the stress is proportional to the strain rate which is the feature of the Newton element; when 0<γ<1, fractional viscoelastic elements are a class of nonlinear elements characterized as intermediate between elastic and Newtonian elements; when γ>1, the more complex nonlinear mechanical behavior of the accelerated rheology can be expressed.

By adjusting the value of *η_i_* and γ*_i_* of the two fractional viscoelastic elements in Figure 4, the fractional viscoelastic element can be flexibly changed between elasticity, viscoelasticity, and viscosity. Viscous elements have a richer combinatorial form and greater generality compared with single viscoelastic elements or viscoelastic elements combined with linear elements. Its specific constitutive equations are derived as follows:

(2) $$\sigma =\sigma_{1}+\sigma_{2}$$

(3) $$\varepsilon =\varepsilon_{1}(t)=\varepsilon_{2}(t)$$

where *σ* and *ε* are the total stress and strain of the viscoelastic element, respectively; *σ*_1_, *ε*_1_ and *σ*_2_, *ε*_2_ represent the stress and strain of viscoelastic element 1 and element 2, respectively.

According to Equation (1):

(4) $$\sigma_{1}=\eta_{1}\frac{d^{\gamma_{1}}\varepsilon_{1}\left(t\right)}{dt^{\gamma_{1}}}$$

(5) $$\sigma_{2}=\eta_{2}\frac{d^{\gamma_{2}}\varepsilon_{2}\left(t\right)}{dt^{\gamma_{2}}}$$

It can be derived from Equation (1) to Equation (5):

(6) $$\sigma =\eta_{1}\frac{d^{\gamma_{1}}\varepsilon_{1}\left(t\right)}{dt^{\gamma_{1}}}+\eta_{2}\frac{d^{\gamma_{2}}\varepsilon_{2}\left(t\right)}{dt^{\gamma_{2}}}$$

Equation (7) can be obtained by performing the Laplace transformation on both sides of Equation (6):

(7) $$\tilde{\sigma }\left(s\right)=\eta_{1}\left[s^{\gamma_{1}}{\tilde{\varepsilon }}_{1}\left(s\right)-s^{\gamma_{1}-1}\varepsilon_{1}\left(0\right)\right]+\eta_{2}\left[s^{\gamma_{2}}{\tilde{\varepsilon }}_{1}\left(s\right)-s^{\gamma_{2}-1}\varepsilon_{1}\left(0\right)\right]$$

Assuming that the value of the stress *σ* applied is constant *σ*_0_, and considering the initial state *ε*_1_(0) = 0, Equation (7) can be transformed into Equations (8) and (9).

(8) $$\frac{\sigma_{0}}{s}=\left(\eta_{1}s^{\gamma_{1}}+\eta_{2}s^{\gamma_{2}}\right){\tilde{\varepsilon }}_{1}\left(s\right)$$

(9) $${\tilde{\varepsilon }}_{1}\left(s\right)=\frac{\sigma_{0}}{s\left(\eta_{1}s^{\gamma_{1}}+\eta_{2}s^{\gamma_{2}}\right)}$$

To obtain the inverse Laplace transform of Equation (9), the definition of the Mittag–Leffler function is as follows:

(10) $$E_{a,b}\left(z\right)=\overset{\infty }{\underset{j=0}{\sum }}\frac{z^{j}}{\Gamma \left(ak+b\right)},\;a>0,\;b>0$$

where Γ() is the Gamma function, defined as:

(11) Γ(z)=∫0∞e−ttzdt, Re(z)>0

According to the Laplace transformation formula of the Mittag–leffler function:

(12) ∫0∞e-pttak+b-1Ea,b(k)(nta)dt=k!pa-b(pa−n)k+1, Re(s)>|n|1a

where $E_{a,b}^{\left(k\right)}\left(nt^{a}\right)$ is the *k*th derivative of the Mittag–leffler function. So, the Laplace inverse transformation of ${\tilde{\varepsilon }}_{1}\left(s\right)$ can be derived, and contacting with the Equation (3), the result is obtained as Equation (13).

(13) $$\varepsilon (t)=\frac{\sigma_{0}t^{\gamma_{2}}}{\eta_{2}}E_{\gamma_{2}-\gamma_{1},\gamma_{2}+1}\left(-\frac{\eta_{1}}{\eta_{2}}t^{\gamma_{2}-\gamma_{1}}\right)$$

This formula is the constitutive model of the fractional viscoelastic element proposed in this paper. For convenience of application, the first two terms of the Mittag–Leffler function are expressed approximately, so that Equation (13) can be simplified as follows:

(14) $$\varepsilon \left(t\right)=\frac{\sigma_{0}t^{\gamma_{2}}}{\eta_{2}}\left[\frac{1}{\Gamma \left(\gamma_{2}+1\right)}+\frac{-\frac{\eta_{1}}{\eta_{2}}t^{\gamma_{2}-\gamma_{1}}}{\Gamma \left(2\gamma_{2}-\gamma_{1}+1\right)}\right]$$

## 4. Nonlinear Creep Constitutive Model of Asphalt Mixture

Due to the presence of asphalt binder, asphalt mixtures can essentially be classified as a type of organic–inorganic composite material with nonlinear viscoelastic-plastic mechanical characteristics. It is often assumed that viscoelastic-plastic materials undergo viscoelastic creep deformation only at low stress. However, during creep beyond the yield strength of the material, as described in Section 1, viscoelastic strain continues to occur in the asphalt mixture. Moreover, a mechanism different from viscoelastic deformation prior to yielding occurs due to the effect of plastic deformation. This means that during the subsequent yielding phase of the asphalt mixture, two types of deformation coexist: viscoelastic and viscoplastic deformation.

**Figure 5:**
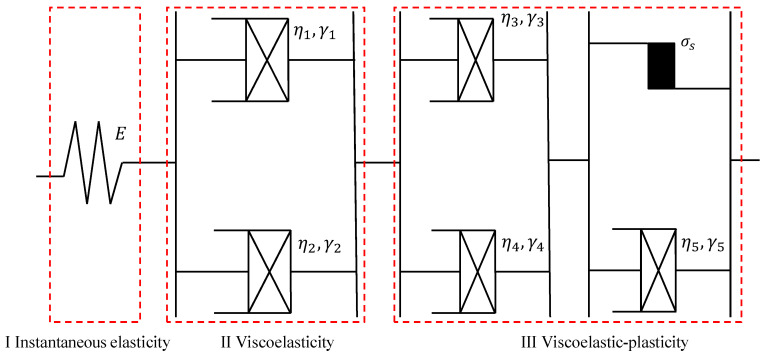
Nonlinear creep constitutive model.

Based on the above views, in this paper, a creep constitutive model of asphalt mixture referring to the Burgers model and the Nishihara model is established, as shown in Figure 5. In the model, the instantaneous elastic strain is represented by a Hooke element, the viscoelastic strain is represented by a viscoelastic element, and the viscoelastic-plastic strain is represented by a viscoelastic element attached to a fractional viscoplastic element. The specific constitutive equations are derived as follows.

For the creep process of asphalt mixtures, when applying the constant stress *σ*_0_, the different components of the strain responses of the nonlinear creep constitutive model can be described as follows:

(1) Instantaneous elasticity

The constitutive equation of the instantaneous elastic model can be obtained from the generalized Hooke law:

(15) $$\varepsilon_{Ⅰ}=\frac{\sigma_{0}}{E}$$

where *E* is the elastic modulus that is corrected considering the amount of plastic deformation caused by the micro-defects existing in the initial structure.

(2) Viscoelasticity

Applying the derivation results of the fractional viscoelastic element constitutive equation in Section 3, the constitutive equation of the viscoelastic model (part II of nonlinear creep constitutive model) is obtained, denoted as Equation (16).

(16) $$\varepsilon_{Ⅱ}\left(t\right)=\frac{\sigma_{0}t^{\gamma_{2}}}{\eta_{2}}\left[\frac{1}{\Gamma \left(\gamma_{2}+1\right)}+\frac{-\frac{\eta_{1}}{\eta_{2}}t^{\gamma_{2}-\gamma_{1}}}{\Gamma \left(2\gamma_{2}-\gamma_{1}+1\right)}\right]$$

where $\varepsilon_{\mathrm{II}}$ is the strain in part II of nonlinear creep constitutive model.

(3) Viscoelastic-plasticity

The strain in part III of the nonlinear creep constitutive model consists of two parts, related by a series relation: the viscoelastic strain due to the effects of plastic deformation and the viscoplastic strain in the subsequent strain-yielding phase.

The constitutive equation of the viscoelastic element in the viscoplastic element is expressed as Equation (17):

(17) $$\sigma_{5}=\eta_{5}\frac{d^{\gamma_{5}}\varepsilon_{5}\left(t\right)}{dt^{\gamma_{5}}}$$

where *σ*_5_ and *ε*_5_(*t*) are the stress and strain on the viscoelastic element, respectively; *η*_5_ is the parameter related to material properties; *γ*_5_ is the corresponding derivative order.

Assuming that the yield stress of the material is *σ_s_*, the viscoplastic constitutive equation of the nonlinear creep constitutive model can be written as follows:

(18) $$\sigma_{5}=\langle \sigma_{0}-\sigma_{s}\rangle =\eta_{5}\frac{d^{\gamma_{5}}\varepsilon_{5}\left(t\right)}{dt^{\gamma_{5}}}$$

where 〈〉 is the Heaviside function. When $\sigma_{0}-\sigma_{s}\leq 0$, the value is 0; when $\sigma_{0}-\sigma_{s}\geq 0$, the value is $\sigma_{0}-\sigma_{s}$.

Applying the Laplace transformation to Equation (18), then taking the inverse Laplace transform, the constitutive equation of the viscoplastic element can be obtained:

(19) $$\varepsilon_{5}\left(t\right)=\frac{\langle \sigma_{0}-\sigma_{s}\rangle t^{\gamma_{5}}}{\eta_{5}\Gamma \left(\gamma_{5}+1\right)}$$

In order to characterize the Viscoelastic–Viscoplastic coexistence mechanism during creep, a fractional viscoelastic element with different parameters from the viscoelastic constitutive equation in part II is taken in the model. According to Equation (16), the strain can be expressed as follows.

(20) $$\varepsilon^{\prime }\left(t\right)=\frac{\langle \sigma_{0}-\sigma_{s}\rangle t^{\gamma_{4}}}{\eta_{4}}\left[\frac{1}{\Gamma \left(\gamma_{4}+1\right)}+\frac{-\frac{\eta_{3}}{\eta_{4}}t^{\gamma_{4}-\gamma_{3}}}{\Gamma \left(2\gamma_{4}-\gamma_{3}+1\right)}\right]$$

where $\varepsilon^{\prime }\left(t\right)$ is the strain representing the coexistence mechanism of viscoelasticity and viscoplasticity during the third phase; *η*_3_ and *η*_4_ are the material parameters of two viscoelastic elements; *γ*_3_ and *γ*_4_ are the corresponding derivative order.

Combining with Equations (19) and (20), the strain of part III of the nonlinear creep constitutive model is as follows:

(21) $$\varepsilon_{Ⅲ}\left(t\right)=\varepsilon^{\prime }\left(t\right)+\varepsilon_{5}\left(t\right)=\frac{\langle \sigma_{0}-\sigma_{s}\rangle t^{\gamma_{4}}}{\eta_{4}}\left[\frac{1}{\Gamma \left(\gamma_{4}+1\right)}+\frac{-\frac{\eta_{3}}{\eta_{4}}t^{\gamma_{4}-\gamma_{3}}}{\Gamma \left(2\gamma_{4}-\gamma_{3}+1\right)}\right]+\frac{\langle \sigma_{0}-\sigma_{s}\rangle t^{\gamma_{5}}}{\eta_{5}\Gamma \left(\gamma_{5}+1\right)}$$

Combining with Equations (15), (16) and (21), the constitutive equation of the creep process constructed is obtained, which is divided into two situations specifically, namely:

(22) $$\{\begin{array}{c} \varepsilon =\varepsilon_{Ⅰ}+\varepsilon_{Ⅱ}=\frac{\sigma_{0}}{E}+\frac{\sigma_{0}t^{\gamma_{2}}}{\eta_{2}}[\frac{1}{\Gamma \left(\gamma_{2}+1\right)}-\frac{\frac{\eta_{1}}{\eta_{2}}t^{\gamma_{2}-\gamma_{1}}}{\Gamma \left(2\gamma_{2}-\gamma_{1}+1\right)}],\;\;\;\;\;\;\;\sigma_{0}<\sigma_{s}\\ \varepsilon =\varepsilon_{Ⅰ}+\varepsilon_{Ⅱ}+\varepsilon_{Ⅲ}=\frac{\sigma_{0}}{E}+\frac{\sigma_{0}t^{\gamma_{2}}}{\eta_{2}}\left[\frac{1}{\Gamma \left(\gamma_{2}+1\right)}-\frac{\frac{\eta_{1}}{\eta_{2}}t^{\gamma_{2}-\gamma_{1}}}{\Gamma \left(2\gamma_{2}-\gamma_{1}+1\right)}\right]\;\;\;\;\;\;\;\;\;\;\;\\ +\frac{\langle \sigma_{0}-\sigma_{s}\rangle t^{\gamma_{4}}}{\eta_{4}}\left[\frac{1}{\Gamma \left(\gamma_{4}+1\right)}-\frac{\frac{\eta_{3}}{\eta_{4}}t^{\gamma_{4}-\gamma_{3}}}{\Gamma \left(2\gamma_{4}-\gamma_{3}+1\right)}\right]+\frac{\langle \sigma_{0}-\sigma_{s}\rangle t^{\gamma_{5}}}{\eta_{5}\Gamma \left(\gamma_{5}+1\right)},\;\;\;\;\sigma_{0}\geq \sigma_{s} \end{array}$$

## 5. Model Parameters Determination and Verification

### 5.1. Determination Method for Model Parameters

In order to obtain the model parameters, the constitutive equation can be simplified as follows:

$$\{\begin{array}{c} \varepsilon =\sigma_{0}\left(\frac{1}{E}+\frac{t^{\gamma_{2}}}{a}-bt^{2\gamma_{2}-\gamma_{1}}\right)\;\;\;\;\;\;\;\;\;\;\;\;\;\;\;\;\;\;\sigma_{0}<\sigma_{s}\;\;\;\;\;\left(23a\right)\\ \varepsilon =\sigma_{0}\left(\frac{1}{E}+\frac{t^{\gamma_{2}}}{a}-bt^{2\gamma_{2}-\gamma_{1}}\right)+\langle \sigma_{0}-\sigma_{s}\rangle \left(ct^{\gamma_{4}}-dt^{2\gamma_{4}-\gamma_{3}}+ft^{\gamma_{5}}\right)\;\;\;\;\;\sigma_{0}\geq \sigma_{s}\;\;\;\;\;(23b) \end{array}$$

where $a=\eta_{2}\Gamma \left(\gamma_{2}+1\right)$, $b=\frac{\eta_{1}}{\eta_{2}{}^{2}\Gamma \left(2\gamma_{2}-\gamma_{1}+1\right)}$, $c=\frac{1}{\eta_{4}\Gamma \left(\gamma_{4}+1\right)}$, $d=\frac{\eta_{3}}{\eta_{4}\Gamma \left(2\gamma_{4}-\gamma_{3}+1\right)}$, $f=\frac{1}{\eta_{5}\Gamma \left(\gamma_{5}+1\right)}$.

When *σ*_0_ is less than *σ*_s_, the Equation (23a) is used to fit the test data with the L-M algorithm and the corresponding parameter *E*, *a*, *b*, *γ*_1_, *γ*_2_ can be obtained directly. Then *η*_1_, *η*_2_ can be obtained by the conversion formulas.

When *σ*_0_ is more than *σ*_s_, segmented fitting is required. According to the material creep test data, the first term of the corresponding constitutive equation in Equation (23b) is fitted by using the test data from 0 to *t*_1_ corresponding to the strain dividing point. The corresponding parameter *E*, *a*, *b*, *γ*_1_, *γ*_2_ is obtained using the method applied in the condition $\sigma_{0}<\sigma_{s}$. In this case, the instantaneous elastic strain and viscoelastic strain of the creep model can be obtained by fitting the results data. However, considering that the viscoelastic strain will not disappear and can continue to develop in the subsequent yield phase, the viscoelastic-plastic strain should be as Equation (24).

(24) $$\varepsilon_{Ⅲ}=\varepsilon -(\varepsilon_{Ⅰ}+\varepsilon_{Ⅱ}),t_{1}\leq t\leq t^{\prime }$$

**Figure 6:**
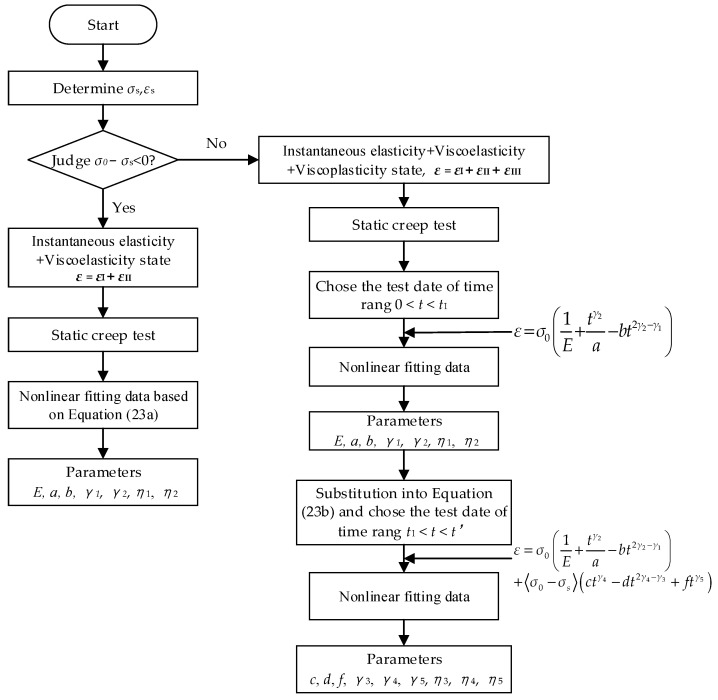
Model parameter determination flowchart.

At this time, the obtained parameter values are fixed, and the viscoelastic-plastic strain data in the creep process are calculated using Equation (24), then the second term of the corresponding constitutive equation in Equation (23b) is applied to fit. The parameters *c*, *d*, *f*, *γ*_3_, *γ*_4_, *γ*_5_ are obtained, and *η*_3_, *η*_4_, *η*_5_ are obtained by the conversion formulas. The process of determining model parameters is shown in Figure 6.

If the yield stress *σ*_s_ is known, the uniaxial compression test is not carried out; namely, the yield strain cannot be determined. For this case, an optimization algorithm was proposed that can quickly determine the yield strain throughout creep without the need for uniaxial compression tests and improve the efficiency of the study.

By setting the number of measuring points on the whole curve as N, and the time corresponding to the Nth measuring point as *t_n_*, then the minimum error between the fitting results and the test results is taken as the target to determine the strain yield point. The optimized model established is shown in Equation (25):

(25) $$\begin{array}{l} \min f=\sum_{i=1}^{n}{\left\{\frac{\sigma_{0}}{E}+\frac{\sigma_{0}t_{i}{}^{\gamma_{2}}}{\eta_{2}}\left[\frac{1}{\Gamma (\gamma_{2}+1)}+\frac{-\frac{\eta_{1}}{\eta_{2}}t_{i}{}^{\gamma_{2}-\gamma_{1}}}{\Gamma (2\gamma_{2}-\gamma_{1}+1)}\right]-\varepsilon_{i}\right\}}^{2}\\ +\sum_{j=n+1}^{N}{\left\{\begin{array}{l} \frac{\sigma_{0}}{E}+\frac{\sigma_{0}t_{j}{}^{\gamma_{2}}}{\eta_{2}}\left[\frac{1}{\Gamma (\gamma_{2}+1)}+\frac{-\frac{\eta_{1}}{\eta_{2}}t_{j}{}^{\gamma_{2}-\gamma_{1}}}{\Gamma (2\gamma_{2}-\gamma_{1}+1)}\right]+\\ \frac{\sigma_{0}-\sigma_{s}t_{j}{}^{\gamma_{4}}}{\eta_{4}}\left[\frac{1}{\Gamma (\gamma_{4}+1)}+\frac{-\frac{\eta_{3}}{\eta_{4}}t_{j}{}^{\gamma_{4}-\gamma_{3}}}{\Gamma (2\gamma_{4}-\gamma_{3}+1)}\right]+\frac{\sigma_{0}-\sigma_{s}t_{j}{}^{\gamma_{5}}}{\eta_{5}\Gamma (\gamma_{5}+1)}-\varepsilon_{j} \end{array}\right\}}^{2} \end{array}$$

**Figure 7:**
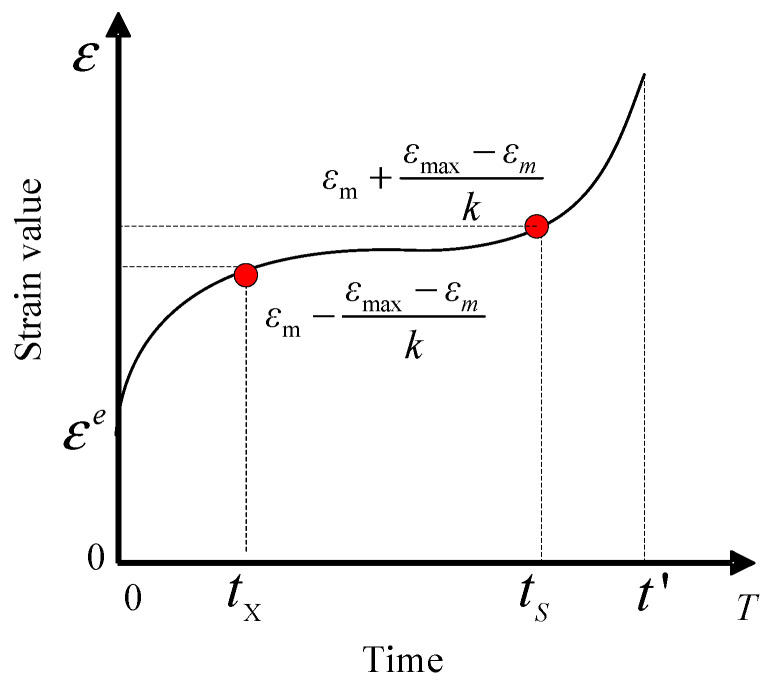
Yield strain determination method.

In the test curve of Figure 7, the yield strain appears in the surrounding region of strain transforming, so simplifying the calculation method of Equation (25) is as follows.

In the range 0 to *t_n_*, first, the forward difference of the strain (the time interval is only related to the test frequency and is a constant more than 0) can be calculated and the time tm corresponding to the minimum difference value and strain *ε_m_* can be determined; second, the possible range of yield strain as Equation (26) can be determined.

(26) $$\varepsilon_{m}-\frac{\varepsilon_{\max}-\varepsilon_{m}}{k}\leq \varepsilon_{s}\leq \varepsilon_{m}+\frac{\varepsilon_{\max}-\varepsilon_{m}}{k}$$

where *ε*_max_ is the maximum strain measured in Figure 6 and *k* is the empirical coefficient. The smaller the value, the larger the range determined, and the longer the optimization time. The recommended value ranges from 3 to 5 as the actual fitting experience.

Solving Equation (25) could use some intelligent optimization algorithms such as the Genetic Algorithm or the Ant Colony Algorithm. The optimized results not only include yield strain point and corresponding time, but also include specific fitting parameters.

### 5.2. Model Verification

Two types of test data are adopted for verification. One is the short-term creep test of CA mortar in the laboratory, and the other is the test data from the references [29,38].

(1) Laboratory creep test

CA mortar is the composition material of the ballastless tracks for high-speed railways. According to Xie’s test research [39], it is known that under the condition of small stress, it takes 3 years or more to obtain the complete creep process of CA mortar. So, the short-time creep test of CA mortar in laboratory is carried out based on Xu’s research [40].

The test of raw materials contain the early strength of Portland cement (P·II 52.5R), fine sand (the maximum particle size not exceeding 1.18 mm), water, SBS-modified emulsified asphalt (the physical properties showed in Table 1), water reducer, defoamer (silicone defoamer), aluminum powder (mass fraction ≥ 85%), and the other additives (early strength agent). The mix proportion of CA mortar is shown in Table 2, where the actual weights of the materials can be weighed according to the proportional relationship shown in the table.

The preparation of CA mortar material: Firstly, pour water, SBS emulsified asphalt, and water reducing agent into a mixer and mix at a speed of 60 r/min for 30 s. Then, add cement, fine sand, defoamer, and aluminum powder, mix at a speed of 60 r/min for 60 s, then mix at a speed of 120 r/min for 180 s, and then mix at a speed of 60 r/min for 60 s. The preparation of CA mortar is complete.

**Figure 8:**
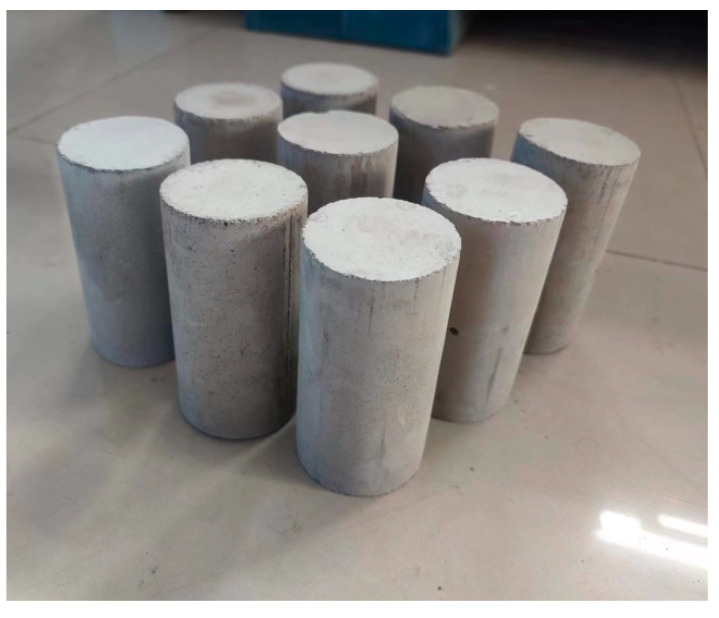
CA mortar specimens.

The preparation process of the test was:

- Pouring the freshly mixed CA mortar into cylindrical specimens with the size of Φ50 × 100 mm;
- Placing in the environment box with relative humidity of (65 ± 5)% and curing for 24 h;
- Removing from the testing mold and curing in standard curing room for 28 days, then the test specimens were completed as shown in Figure 8;
- After completing the above steps, taking out specimens and carrying out the creep loading test of CA mortar.

**Figure 9:**
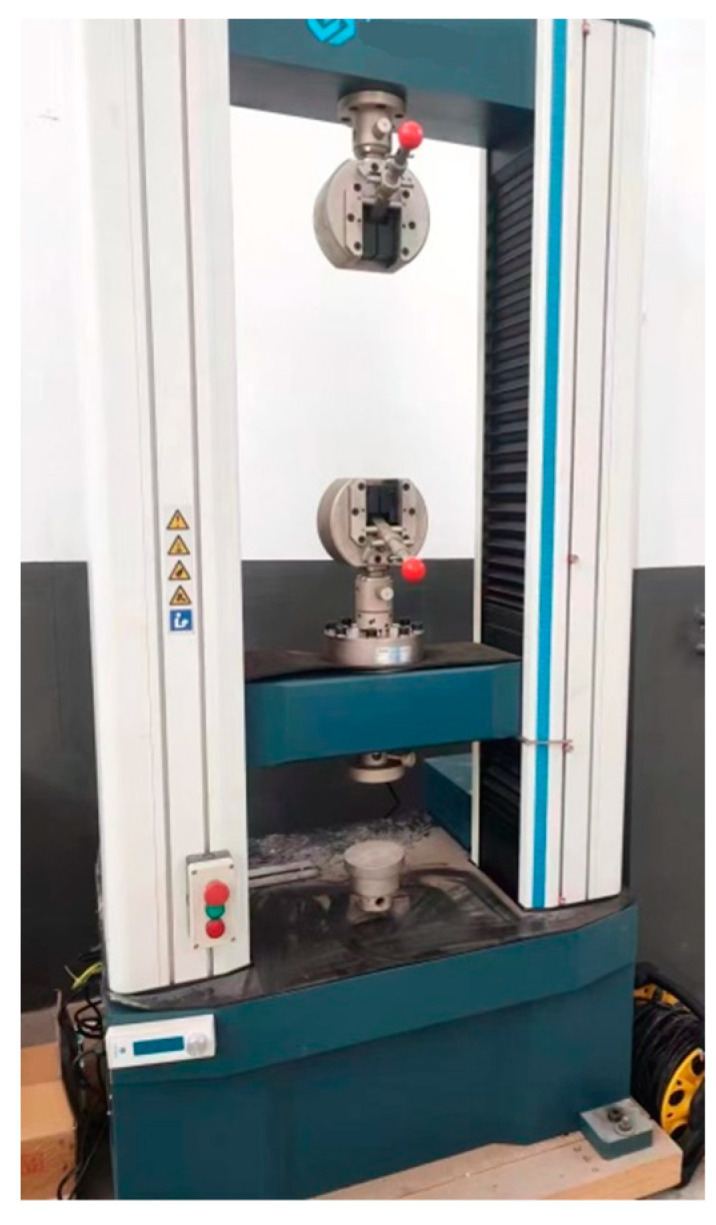
Electronic universal testing machine.

The creep test used the electronic universal testing machine made in China shown in Figure 9. The measurement range of the machine is 50 kN and the accuracy is ±0.5% of the value displayed.

The test was carried out at an environment temperature of 25 °C.

The loading stress levels were set at 0.1 MPa and 0.5 MPa, and the data acquisition frequency was 0.04 Hz. Before starting the normal test, 0.005 MPa should be preloaded to eliminate the gap between the test instrument and the specimen. The stress of 0.005 MPa should be maintained for 1 min and then the set stress level should be loaded quickly and maintained for 1.0 h. In order to ensure the stability of the test data, the test data with large discreteness were removed, and the number of specimens was increased to ensure that each stress level had three groups of qualified data. Then, the data curve changes smoothly for the fitting of the constitutive model.

**Figure 10:**
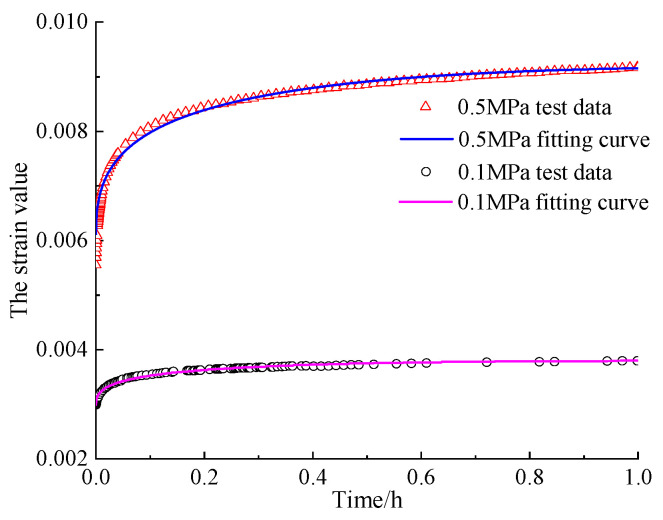
Short-term creep test data and fitting results of CA mortar.

According to the regulation [41] and the compressive strength value of CA mortar measured in the previous experiment, the 28-day compressive strength of CA mortar is 1.8 MPa, and it is pointed out in reference [42] that the recommended yield strength of general viscoelastic materials is 40% of the compressive strength value. So, if the minimum yield value of CA mortar is calculated in accordance with the specification, the yield stress value of CA mortar is 0.72 MPa. Because the stress values applied in the specimens are all smaller than 0.72 MPa, the corresponding equation of the case *σ*_0_ < *σ*_s_ in the established constitutive Equation (23a,b) was used to fit the test data. The test data and model fitting results are shown in Figure 10. The fitting parameters are shown in Table 3. In order to increase the reliability of the model validation process in this article and the impact of different models on the characterization of mechanical properties of the same material, this article compared the short-term creep test data of CA mortar in reference [40]. The specimen size used in this experiment is Φ50 × 50 mm (Φ50 × 100 mm in this article), the test temperature is controlled at 25 °C (the same as the test environment temperature in this article), and the loading stress levels are 0.1 MPa and 0.5 MPa, respectively (the same as the test loading stress size in this article). The loading process also involves preloading with a stress load of 0.005 MPa for 10 min, then quickly applying it to the required loading stress value and maintaining it for 1 h. In reference [40], the Burgers model and the four-element five-parameter model were used to fit experimental data, and parameter E, which also characterizes the instantaneous elastic deformation of the CA mortar creep process, was compared, as shown in Table 4.

Figure 10 shows that the viscoelastic-plastic constitutive model established in this paper can be used to fit the test data under different stresses during the short-term creep process of CA mortar, and the goodness of fit is above 0.99. Meanwhile, compared with the CA mortar elastic model established in the literature in Table 4, it can be seen that the fitting results are consistent under the condition of 0.1 MPa, but the elastic modulus obtained under 0.5 MPa stress is slightly smaller, with 3.33% deviation from Burgers model and 9.58% deviation from the four-element, five-parameter model. On the one hand, it may be caused by different test loading conditions; on the other hand, it is due to the differences in the physical expressions of material deformation by different constitutive models.

(2) Model adaptability verification

**Figure 11:**
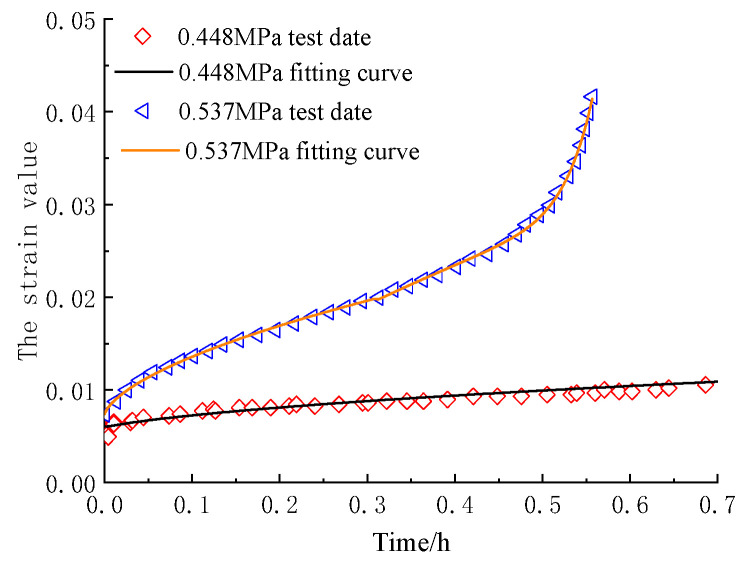
Fitting of creep test results of asphalt mixture.

**Figure 12:**
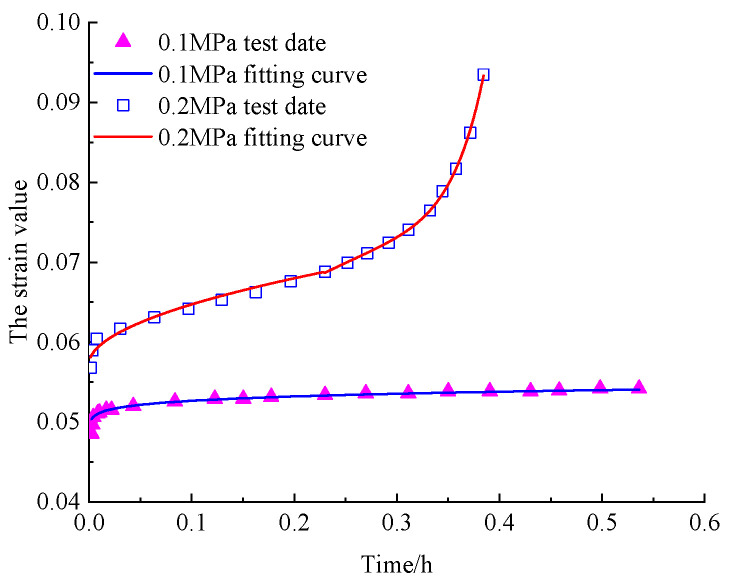
Fitting of creep test results of asphalt sands.

In order to verify the adaptability of the model to asphalt mixture, the creep test data of different components of asphalt mixture in references [29,38] were fitted with the established model, and the fitting results were shown in Figure 11 and Figure 12.

According to the reference corresponding to the test data, the yield strength of asphalt mixture and asphalt sand are 0.358 MPa and 0.05 MPa, respectively. The asphalt mixture in Figure 11 at a stress value of 0.537 MPa and the asphalt sand in Figure 12 at a creep value of 0.2 MPa both demonstrated obvious viscoelastic-plastic deformation phases. Therefore, the test data should be fitted by using the constitutive equation parameter determination method corresponding to the case of *σ*_0_ ≥ *σ*_s_ in Section 5.1. However, the creep deformation rate of asphalt mixture under a stress value of 0.448 MPa and asphalt sand under a stress value of 0.1 MPa was slow, and the strain value did not reach the yield strain value during the test time. So, the test data should be fitted with the first two terms of the constitutive equation corresponding to the case of *σ*_0_ ≥ *σ*_s_.

As the results shown in Figure 11 and Figure 12 and Table 5 demonstrate, the fractional-order creep model established considering the Viscoelastic–Viscoplastic coupling mechanism in this paper has a good fitting effect at different phases of the creep process under different stress conditions. And it also shows that the dividing method based on the strain yield proposed in this paper is also feasible.

## 6. Conclusions

Based on the derivation of the nonlinear creep constitutive model for asphalt mixtures and the experimental validation results, the following conclusions are obtained.

1. The strain yield point of viscoelastic plastic materials is used as the basis for dividing the creep process, solving the problem of strain characteristics at different stages of the creep process. For asphalt mixtures with viscoelastic plastic mechanical characteristics, their complete creep strain properties can be divided into different types including instantaneous elasticity, viscoelasticity, and viscoelastic-plasticity.
2. The coexistence mechanism of multiple strains in the subsequent yield phase of viscoelastic-plastic materials was proposed. This method can provide a new analytical method for studying the coupling effect of viscoelastic viscoplastic mechanical behavior. Based on the theory of fractional calculus, a special fractional order viscoelastic element consisting of two fractional order viscoelastic elements in parallel has been established. It can more flexibly characterize the elastic, viscoelastic, and viscous mechanical behaviors of materials, providing a generalized model reference for the study of material mechanical behavior.
3. Through short-term creep tests of CA mortar indoors and fitting results with multi stress creep tests of asphalt mixtures composed of different materials in the literature, it is shown that under small stress, the creep process of asphalt mixtures exhibits more viscoelastic deformation. Under large stress, the creep process of asphalt mixtures has a complete three-phase property. When the strain value exceeds the yield strain, plastic deformation occurs.
4. The creep constitutive model of asphalt mixture established in this paper has excellent fitting accuracy for the creep process of CA mortar, asphalt mixture (applied to pavement engineering), and asphalt sand. The minimum fitting correlation values R^2^ are 0.9976, 0.981, and 0.979, respectively. The fitting results also indicate that the model proposed in this paper has good adaptability for different types of asphalt matrix mixtures with creep properties.
5. This article only verified the established model through the creep behavior of asphalt matrix mixtures and whether it is suitable for the creep behavior of other materials still needs to be studied. At the same time, when analyzing the creep behavior of materials using the model established in this paper, it is necessary to obtain the yield strain value of the material under corresponding conditions.

## Figures and Tables

**Table 1:** Properties of asphalt emulsion.

De-Emulsification Speed	Evaporation Residue			
	Residue Content/%	Penetration Index/ (0.1 mm)	Softening Point/°C	Ductility (25 °C)/cm
Slow-breaking	58.5	62.3	45.5	130

**Table 2:** **Q**uality mix proportion of CA mortar.

Material	Cement	Fine Sand	Emulsified Asphalt	Water	Water Reducer	Defoamer	Aluminum Powder
Weight parts	366.7	733.3	515	50	12.5	1.5	0.05

**Table 3:** Fitting values of model parameters under different stress levels.

Stress/MPa	E/MPa	*γ*_1_	*γ*_2_	*a*	*b*	R-Squared
0.1	33.46	0.5047	0.5095	1.1646	0.8505	0.9985
0.5	81.7285	0.5335	0.5364	0.998	0.9959	0.9976

**Table 4:** Parameters of CA mortar creep test model in reference [40].

Stress/MPa	Elastic Modulus/MPa (Burgers Model)	Elastic Modulus/MPa (Four-Element Five-Parameter Model)
0.1	33.326	35.49
0.5	84.549	90.39

**Table 5:** Fitting results of creep test parameters of different asphalt mixtures by this model.

Data Sources	Stress	*E*	*γ*_1_	*γ*_2_	*a*	*b*	*γ*_3_
Reference [29]	0.448	69.053	0.385	0.382	0.952	1.037	
	0.537	72.264	0.483	0.478	0.811	1.186	35.492
Reference [38]	0.1	2.799	0.0472	0.0471	0.822	1.027	
	0.2	3.488	0.527	0.526	0.9465	0.9318	26.948
**Date Source**	**Stress**	***γ*_4_**	***γ*_5_**	***c***	***d***	***f***	**R^2^**
Reference [29]	0.448	-	-	-	-	-	0.981
	0.537	18.02	0.5464	2625.16	0.737	1.154	0.999/0.998
Reference [38]	0.1	-	-	-	-	-	0.979
	0.2	13.047	0.148	30233.418	0.010	1.148	0.980/0.998

## Data Availability

Because the research data in this paper involves the interests and privacy of some enterprise projects, it is not convenient for publication at the moment.
